# Agroindustrial By-Products as a Source of Biostimulants Enhancing Responses to Abiotic Stress of Horticultural Crops

**DOI:** 10.3390/ijms25063525

**Published:** 2024-03-20

**Authors:** Javier Zuzunaga-Rosas, Monica Boscaiu, Oscar Vicente

**Affiliations:** 1 Institute for the Conservation and Improvement of Valencian Agrodiversity (COMAV), Universitat Politècnica de València, Camino de Vera s/n, 46022 Valencia, Spain; jazuro@doctor.upv.es (J.Z.-R.); ovicente@upvnet.upv.es (O.V.); 2 Mediterranean Agroforestry Institute (IAM), Universitat Politècnica de València, Camino de Vera s/n, 46022 Valencia, Spain

**Keywords:** ionic stress, osmotic stress, oxidative stress, stress markers, salt tolerance, antioxidant systems, molecular mechanisms, circular economy

## Abstract

Together with other abiotic stresses such as drought and high temperatures, salt stress is one of the most deleterious environmental factors affecting plant development and productivity, causing significant crop yield reductions. The progressive secondary salinisation of irrigated farmland is a problem as old as agriculture but is aggravated and accelerated in the current climate change scenario. Plant biostimulants, developed commercially during the last decade, are now recognised as innovative, sustainable agronomic tools for improving crop growth, yield, plant health and tolerance to abiotic stress factors such as water and soil salinity. Biostimulants are a disparate collection of biological extracts, natural and synthetic organic compounds or mixtures of compounds, inorganic molecules and microorganisms, defined by the positive effects of their application to crops. The growing interest in biostimulants is reflected in the increasing number of scientific reports published on this topic in recent years. However, the processes triggered by the biostimulants and, therefore, their mechanisms of action remain elusive and represent an exciting research field. In this review, we will mainly focus on one specific group of biostimulants, protein hydrolysates, generally produced from agricultural wastes and agroindustrial by-products—contributing, therefore, to more sustainable use of resources and circular economy—and primarily on the consequences of their application on the abiotic stress resistance of horticultural crops. We will summarise data in the scientific literature describing the biostimulants’ effects on basic, conserved mechanisms activated in response to elevated salinity and other abiotic stress conditions, such as the control of ion transport and ion homeostasis, the accumulation of osmolytes for osmotic adjustment, or the activation of enzymatic and non-enzymatic antioxidant systems to counteract the induced secondary oxidative stress. The collected information confirms the positive effects of biostimulants on crop tolerance to abiotic stress by enhancing morphological, physiological and biochemical responses, but also highlights that more work is needed to further establish the molecular mechanisms underlying biostimulants’ effects.

## 1. Introduction

Modern agriculture faces the dual challenge of ensuring global food security and implementing it in a sustainable manner. However, abiotic and biotic stresses make it difficult to achieve these objectives and are becoming significant threats to plant survival, growth and development [1]. Abiotic stresses—such as drought, extreme cold or heat and salinity—have a greater impact on crops’ growth, biomass production and yield than biotic stresses [2]. In terms of salinity stress, roughly 20% and 33% of the world’s land and irrigated land area, respectively, are affected by salinity [3]. Although salinisation is more pronounced in arid and semi-arid climate regions, it is a process that occurs on almost all continents and in a wide range of climates [4]. This situation has worsened over the years in cultivated areas worldwide due to the impact of accelerated climate change [5,6].

In general, “secondary” salinisation—due to human activities—in irrigated cropland is due to the progressive accumulation in the soil of toxic ions dissolved in the irrigation water. The problem is aggravated by the use of low-quality, saline water for irrigation, the excessive use of chemical fertilisers [7,8] or poor soil drainage conditions [9,10]. In cultivated areas close to the sea, an additional problem is seawater intrusion [11], especially when aquifers have been overexploited.

The accumulation of soluble salts in the soil is one of the main reasons for the low productivity of many economically important or high-value crops [7]. Increased salinity in the rhizosphere negatively affects germination rate, vegetative growth, reproductive development and, ultimately, crop yield; it can even result in plant death [12]. High concentrations of soluble salts in the soil profile directly cause physiological drought in plants, i.e., reduced water uptake due to salt accumulation in the root zone [13] and, consequently, osmotic stress and plant dehydration. Excessive uptake and accumulation of Na^+^ and Cl^−^ in the plant cause many physiological disturbances and the disruption of cell metabolic functions due to the toxicity of these ions [13,14]. Excess Na^+^ ions in plant tissues damage the cell membrane and organelles, resulting in cell death [15]. Some of these deleterious effects are mediated by the production of reactive oxygen species (ROS), reduced rate of photosynthesis and scavenging of antioxidants [16]. Therefore, salinity affects plant growth and function through three components: osmotic stress, ion toxicity and oxidative stress [17].

Plants have evolved multiple and complex morphological, physiological, biochemical and molecular responses to survive and adapt to salt stress [18]. They include the biosynthesis of various primary and secondary metabolites that are widely known to be important components of plant defence mechanisms against stressful conditions [19]. These compounds are, for example, osmolytes and osmoprotectants involved in osmotic adjustment, recovery and signalling, stabilisation of proteins and protein complexes in the chloroplast and cytosol and protection of the photosynthetic apparatus, such as sugars, amino acids or polyols [20]; they maintain osmotic balance and cell turgor, prevent cell dehydration, stabilise and protect the tertiary structure of proteins and membranes [21]. Several antioxidant enzymes are also activated, such as superoxide dismutase (SOD), catalase (CAT), ascorbate peroxidase (APX) and other peroxidases or glutathione reductase (GR), and antioxidant metabolites are synthesised, including reduced glutathione (GSH), ascorbate, carotenoids, flavonoids and other phenols [22]. Apart from specific ion toxicity effects, other abiotic stress conditions, such as drought, waterlogging or too high or too low temperatures, also cause osmotic and oxidative stress similar to high salinity and, therefore, trigger the same responses, mediated by the synthesis of osmolytes/osmoprotectants and the activation of antioxidant systems (e.g., [2,16,19]).

Various strategies have been implemented over time to mitigate the negative effect of abiotic stress and increase salt stress tolerance in horticultural crops, including conventional breeding for the development of new varieties, gene cloning and genetic engineering or genome editing [23,24]. However, the complex mechanisms of salinity tolerance and the limited genetic variability amongst crop varieties and their wild relatives regarding this trait have led to limited success in generating commercial crop cultivars tolerant to salt stress [25,26,27].

The so-called plant biostimulants have gained relevance in recent years for their ability to regulate or modify different plant physiological processes, resulting in growth stimulation, increased yields, improved quality traits and enhanced plant responses to abiotic stress [28]. Other advantages are the absence of negative or harmful impacts on plants, humans, animals or the environment, the increase in biodiversity of beneficial microorganisms and the improvement of soil properties [29], gaining the attention of not only scientific but also agroindustrial businesses [30]. The commercial success of these compounds is such that the global market for biostimulants is expected to exceed USD 9.2 × 10^9^ by 2027 [31]. Biostimulants can be produced from different inorganic and organic sources, from microbial (beneficial fungi and bacteria) to non-microbial, which include a wide variety of elements such as extracts of algae and microalgae, humic, fulvic and carboxylic acids, vitamins, amino acids, ascorbic acid, phenolic compounds and protein hydrolysates [29,32].

The biostimulants’ effects on crops, mentioned above, are supported by many published reports; however, the processes triggered by biostimulants and, thus, their mechanisms of action remain elusive and represent a challenging topic of research. The study of biostimulants is too broad a field to be addressed in depth in a single review because of their varied origins and multiple effects. Therefore, this article will primarily focus on a specific group of biostimulants, those produced from hydrolysates of proteins and mainly on the consequences of their application on salt resistance in horticultural crops. Protein hydrolysates, obtained from plant and animal agroindustrial waste and by-products, are particularly valuable from the perspective of a circular economy [33,34,35] in line with the European Union objectives and legislation [36], contributing to improving the productivity of sustainable agricultural systems in the current context of accelerated climate change, to reduce chemical inputs (e.g., fertilisers) and the impact of waste on the environment [37,38].

We will summarise data from the scientific literature describing the effects of protein hydrolysates-based biostimulants on basic, conserved mechanisms activated in response to high salinity. These responses include the control of ion transport and ion homeostasis, the accumulation of osmolytes for osmotic adjustment or the activation of enzymatic and non-enzymatic antioxidant systems to counteract secondary salt-induced oxidative stress and possible changes in gene expression patterns. We will also mention some examples of biostimulants affecting plant responses triggered by other abiotic stress factors and, briefly, their stimulation of plant growth in the absence of stress. The information gathered shed some light on the modes of action of these protein hydrolysates-based biostimulants; however, more work is needed to understand the molecular mechanisms underlying their effects.

## 2. Biostimulants

### 2.1. Definition and Classification

**Figure 1:**
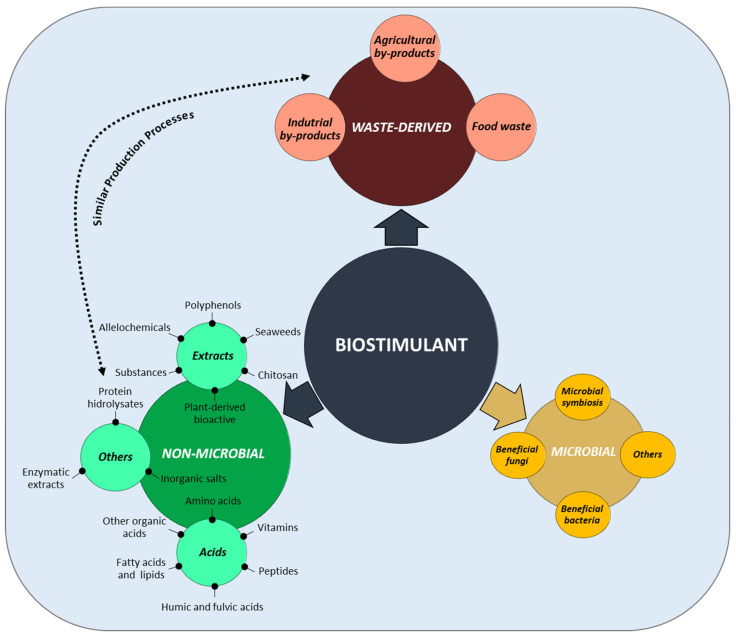
Biostimulant categories, defined according to the sources from which they are produced (Based on European Commission [36]).

Biostimulants have different mechanisms of action and a complex nature, so their definition is still under debate. Du Jardin [30] proposed the following definition, considering biostimulants’ general properties: “A plant biostimulant is any substance or microorganism applied to plants with the aim to enhance nutrition efficiency, abiotic stress tolerance and/or crop quality traits, regardless of its nutrients content. By extension, plant biostimulants also designate commercial products containing mixtures of such substances and/or microorganisms”. Over the years, this definition has gained relevance and has recently been adapted and modified by the European Biostimulants Industry Council (EBIC), considering biostimulants as materials containing substance(s) and/or microorganisms whose function is to stimulate natural processes and mechanisms to benefit nutrient uptake and efficiency, abiotic stress tolerance and crop quality when applied to plants or the rhizosphere, independent of their nutrient content [39]. Du Jardin [30] also classified biostimulants by their content and proposed seven categories: humic and fulvic acids, protein hydrolysates, chitosan and other biopolymers, seaweed extracts and botanicals, beneficial minerals, beneficial bacteria and beneficial fungi. However, this classification has also undergone modifications, and now biostimulants are grouped in the European Union [36] into three categories according to the origin of their active ingredients, as shown in Figure 1: (1) non-microbial biostimulants, including extracts (polyphenols, seaweeds, chitosan, plant-derived bioactive substances and allelochemicals), acids (amino acids, vitamins, peptides, humic and fulvic acids, fatty acids and lipids and other organic acids) and others (protein hydrolysates, enzymatic extracts and inorganic salts); (2) waste-derived biostimulants (industrial by-products, agricultural by-products and food waste); and (3) microbial biostimulants (beneficial fungi, beneficial bacteria, microbial symbiosis and others). However, there are also other forms of classification, such as grouping biostimulants into only two categories, non-microbial and microbial [40], with waste-derived or by-product-derived biostimulants [41] included within the non-microbial ones, as they share similar production or production processes in addition to their nature.

### 2.2. Mechanisms of Action: Function and Defence against Abiotic Stress

Biostimulants offer a potentially novel approach for regulating and modifying physiological processes in plants to mitigate stress-induced adverse effects, stimulate growth and increase yield, thus promoting productivity optimisation [28].

However, the mode of action of a particular biostimulant—the morphological and physiological changes it induces in plants—and the molecular mechanisms underlying its effects are not yet clearly defined according to the type, origin or category of that biostimulant [42]. Several studies (e.g., [43,44,45,46]) have reported that biostimulants belonging to different categories share similar modes of action and mechanisms in increasing plant tolerance to abiotic stresses. On the other hand, one specific biostimulant generally activates several response mechanisms, leading to multiple beneficial effects.

Another aspect to be considered is the biostimulants’ potential to induce transgenerational plasticity and metabolite accumulation within plants, which is related to their mechanisms of action since it has been reported that biostimulants succeed in regulating molecular mechanisms triggering an increased expression of genes and proteins related to stress [42]. In addition, biostimulants may have different effects according to the plant species and the type of stress involved, and indeed, the type of biostimulant used or their application method, foliar, in the soil or through the irrigation system. Along the same lines, in addition to the biostimulants’ effects on abiotic stress responses, a stimulating effect on plant growth in the absence of stress has been observed in many cases [34,42,45], probably due to a general improvement in photosynthesis and primary metabolism.

For instance, Kałuzewicz et al. [47] assessed how biostimulants affected two cultivars of *Brassica oleracea* var. *italica* against drought stress regarding their chlorophyll content and other physiological characteristics. Applications of biostimulants were made to the soil and the leaves, in both cases increasing resistance to drought and producing positive effects in terms of photosynthetic rate, stomatal conductance, internal CO_2_ concentration and transpiration rate; however, the effects varied depending on the cultivar.

Likewise, Rady et al. [48] evaluated the effect on *Phaseolus vulgaris* plants subjected to salt stress of a biostimulant based on a plant extract that was used for seed soaking and then for foliar applications. As a result of seed soaking, plants under saline conditions showed greater shoot length, a higher number of leaves and pods and increased photosynthetic pigment contents compared to the non-treated controls. After the foliar applications, the plants achieved higher growth and yield, higher contents of antioxidant compounds such as carotenoids or sugars and the activation of antioxidant enzymes, leading to a decrease in reactive oxygen species (ROS) and a higher membrane stability index.

Biostimulants cannot be defined as fertilisers because they do not provide nutrients directly to plants. However, they can facilitate the nutrient acquisition by supporting metabolic processes in soil and plants, e.g., by mobilising elements in the rhizosphere or by developing new nutrient acquisition pathways [49]. Unlike fertilisers or pesticides, whose active ingredients are usually specific molecules with a clearly defined mechanism of action, the use of biostimulants manifests multiple effects, as they may contain different active compounds in their complex composition, making their mechanism(s) of action challenging to elucidate. Therefore, biostimulants require further research and evaluation in the field, even though their beneficial effects on tolerance against abiotic stresses have been well-established in numerous studies. In addition, biostimulants differ from fertilisers, in that they elicit cellular and adaptive responses, regardless of the presence of nutrients in their formulations [50]; therefore, they are used as complements but not substitutes for fertilisers.

**Figure 2:**
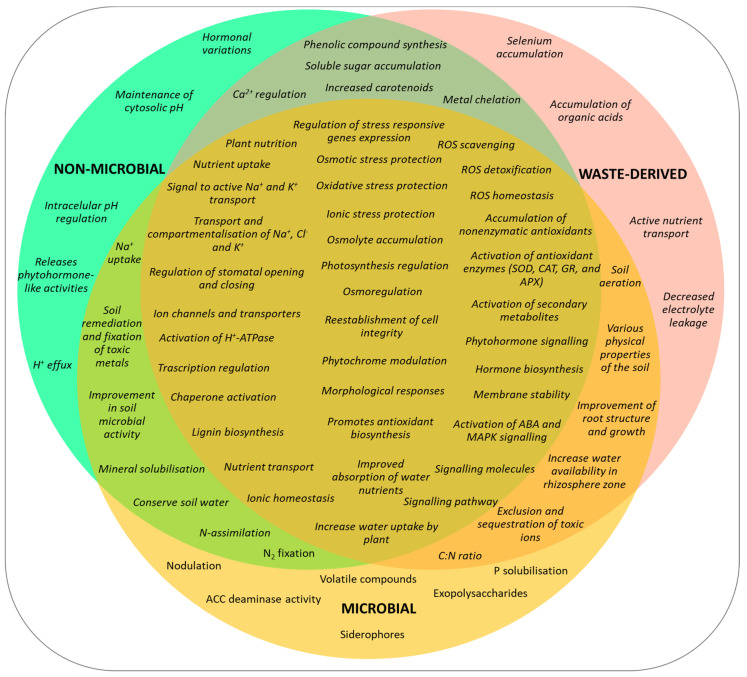
Venn diagram of the mechanisms induced by different biostimulants, classified according to their origin—microbial, non-microbial and waste-derived—contributing to enhancing plant tolerance to abiotic stress, highlighting the extensive overlapping of the effects of biostimulants belonging to the three groups. Prepared from information in references [42,58].

Efforts have been made over time to elucidate the mechanisms of action of biostimulants (e.g., [51,52,53,54,55,56,57]), and significant advances have been achieved through the use of physiological and biochemical approaches, and particularly omics techniques, including genomics, transcriptomics, proteomics and metabolomics. However, our knowledge of these mechanisms is still limited, and these studies represent a great challenge for future research. As shown in the scientific literature, when considering the biostimulant-dependent improvement of abiotic stress responses, there is a considerable overlap of the observed effects and mechanisms of action of biostimulants belonging to the different groups—microbial, non-microbial and waste-derived biostimulants. These overlapping effects include, amongst many others, regulating the expression of stress-responsive genes or activating phytohormone—particularly abscisic acid (ABA)—or MAP kinase signalling and also, improving photosynthesis and water and nutrient uptake by the plants and protecting them against ionic toxicity by controlling Na^+^ and Cl^−^ transport and compartmentalisation and K^+^ homeostasis. Furthermore, all biostimulant types contribute to osmotic balance by promoting osmolyte accumulation and counteract oxidative stress by increasing antioxidant enzyme (e.g., SOD, CAT, GR and APX) activities and activating the synthesis of antioxidant compounds. These and many other biostimulants’ effects are summarised in Figure 2, which provides an overview of the multiple physiological and biochemical responses induced in the plants, as well as changes observed in the soil, by application of the different biostimulant types.

## 3. Protein Hydrolysates

### 3.1. Definition and Agronomic Importance

Protein hydrolysate biostimulants are of growing interest in sustainable agriculture due to their significant effect on increasing crop yields under abiotic stress conditions [59]. These complex mixtures of amino acids and oligopeptides are produced under controlled conditions, either by chemical hydrolysis, enzymatic hydrolysis or a combination of both, from the proteins of plant or animal origin contained in residual sources, by-products or wastes of agroindustrial processes [60,61,62,63,64]. They are usually readily available at an affordable cost due to the abundance of the source raw materials [65,66]. Moreover, from an environmental, economic and social point of view, their production is relevant as they provide a potential solution to the problem of disposal or management of waste, which is often toxic or causes soil and water contamination [67,68]. Production of this kind of biostimulant also represents a paradigmatic example of circular economy.

### 3.2. Effects and Mechanisms on Plant Abiotic Stress Tolerance

Many studies (e.g., [69,70,71,72,73,74,75,76,77,78]) have reported beneficial effects of the application of protein hydrolysate biostimulants on crop growth, yield and product quality (e.g., in tomato, spinach, lettuce, celery, melon, chickpea, maize or beans). For example, Testani et al. [79] in *Capsicum annuum* and Choi et al. [80] in *Solanum lycopersicum* and *Lactuca sativa* observed that protein hydrolysates increased nutrient uptake, especially nitrogen, by acting directly on enzymes of nitrogen and carbon metabolism, such as glutamine synthetase, glutamate synthase, nitrate reductase, nitrite reductase, malate and isocitrate dehydrogenases and citrate synthase; these results are in agreement with those obtained in *Zea mays* plants [81]. On the other hand, Ceccarelli et al. [44] detected phytohormone-like activities in tomato plants, in response to the application of biostimulants based on protein hydrolysates, which strongly stimulated the accumulation of cytokinin, auxin and gibberellin precursors.

The ability of protein hydrolysate biostimulants to activate or enhance defence mechanisms against abiotic stress has also been demonstrated. For example, in tomato plants, when a protein hydrolysate biostimulant was used to mitigate the effects of excess salts in soil and irrigation water, together with growth stimulation, a reduction of toxic ions (Na^+^, Cl^−^) contents and an increase in beneficial cations (K^+^ and Ca^2+^) concentrations were detected in roots and leaves. Compared to control plants not treated with the biostimulant, significant reductions in proline and oxidative stress markers (hydrogen peroxide and malondialdehyde) contents and antioxidant enzyme (catalase, glutathione reductase and superoxide dismutase) activities were also observed [82]. Therefore, the biostimulant reduced the level of osmotic, ionic and oxidative stress induced in the plants under high salinity conditions.

**Figure 3:**
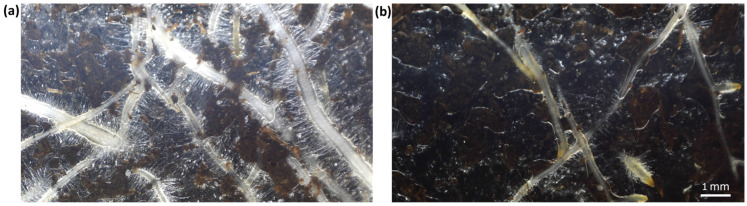
*Lactuca sativa* roots under salt stress conditions (50 mM NaCl). (**a**) Plants treated with BALOX^®^, a biostimulant based on plant protein hydrolysates and (**b**) control, non-treated plants; see [89].

Likewise, Zhang et al. [83] and Tallarita et al. [84] reported increased levels of secondary antioxidant metabolites in tomato plants treated with protein hydrolysates. These compounds include ascorbic acid, flavonoids and other polyphenols and carotenoids like lycopene, also indicating the activation of antioxidant defence responses against salinity and other abiotic stresses. Furthermore, it has been reported in various crops, such as lettuce, maize, grapevine or tomato, that the treatment with biostimulants based on protein hydrolysates mimicked phytohormone activities similar to auxins and gibberellins [63,69,83,85,86,87,88]. These are only a few examples of the physiological and biochemical effects of applying this type of biostimulant to horticultural crops. Protein hydrolysates have also been shown to positively affect plant morphology. Balox^®^, a commercial biostimulant obtained from rice and oat husks protein hydrolysates containing polyphenols and glycine betaine as additional active components, serves as a good example. This product, applied to the root system of *Lactuca sativa* plants by irrigation, improves tolerance against salt stress by decreasing oxidative stress, promoting growth and improving photosynthesis and primary metabolism. As shown in Figure 3, the enhanced salt tolerance is due, at least in part, to the protection of the root absorption zone from the damage caused under high salinity conditions, favouring the emission of finer roots primarily responsible for nutrient uptake [89].

## 4. Agroindustrial By-Products as a Source of Biostimulant

**Figure 4:**
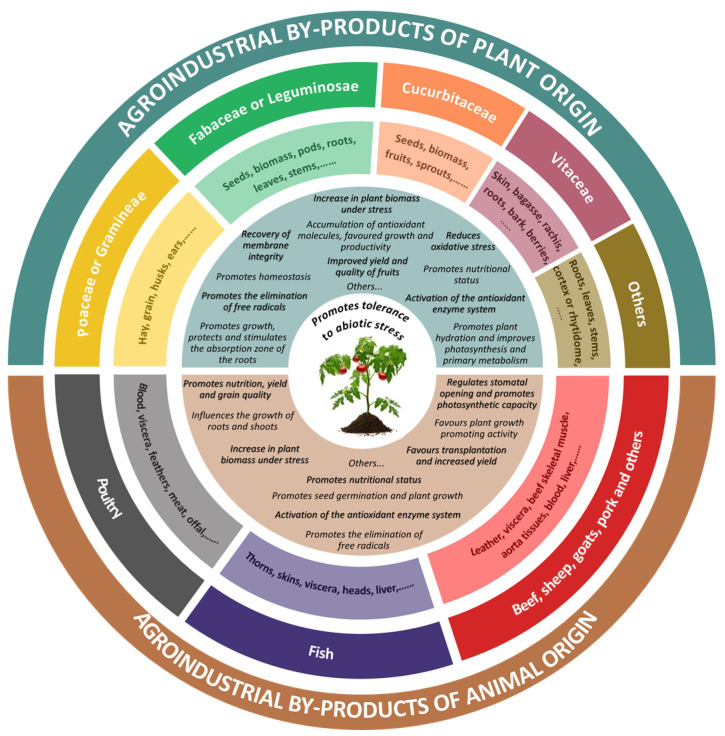
Protein hydrolysates derived from agroindustrial waste of plant or animal origin and their effects on improving crop abiotic stress tolerance. Adapted from Colla et al. [68] and Sun et al. [96].

Chemical or enzymatic hydrolysis of proteins produces amino acids and peptide mixtures from agroindustrial by-products, which can be of vegetable origin (crop by-products: seeds, husks, biomass and fruits) [34,57,84,86,89,90] or derived from animals, e.g., blood, feathers, viscera, bones, skins and other waste products [35,76,81,91,92,93,94]. These mixtures contain bioactive compounds that function as plant biostimulants, improving growth and enhancing abiotic stress tolerance. An overview of these biostimulant sources and their effects enhancing plant abiotic stress tolerance is depicted in Figure 4.

The use of agroindustrial waste or by-products as raw materials for the production of plant biostimulants allows for more efficient use of resources and represents an excellent example of a circular economy, in line with the EU objectives [36]. By improving nutritional efficiency, biostimulants also help reduce the excessive use of chemical fertilisers such as nitrates, contributing to sustainability in agricultural systems [95].

### Effect of Protein Hydrolysates on Plant Abiotic Stress Responses

Many recent reports support the use of biostimulants based on protein hydrolysates of agroindustrial by-products to enhance the responses of horticultural crops to different abiotic stresses. El-Nakhel et al. [78] evaluated a protein hydrolysate derived from plants of the Poaceae family in lettuce plants grown under various salinity conditions and found increased levels of antioxidant metabolites, such as total phenolic acids, flavonoids and lutein, with differences depending on the protein hydrolysate fraction used. Along the same lines, Zuzunaga-Rosas et al. [57] evaluated the effects of the biostimulant Balox^®^, mentioned above, on *Solanum lycopersicum* plants, reporting improved growth under salt stress conditions, and increased foliar levels of photosynthetic pigments and total soluble sugars, not only under stress conditions but also in the absence of salt. Other studies [53] showed favourable effects of a protein hydrolysate on the tolerance to water stress and oxidative imbalance in tomato plants, involving the scavenging of prenyl quinone radicals and hydroxycinnamic amide signalling. Likewise, Sitohy et al. [73] reported that treatment with a pumpkin seed protein hydrolysate mitigated the effects of salt stress in *Phaseolus vulgaris*, by enhancing the antioxidant capacity of the plants, reducing MDA content and also restoring ionic homeostasis.

On the other hand, Wang et al. [35] evaluated an animal-derived biostimulant in tomato plants subjected to drought stress. They reported the activation of stress defence mechanisms, including the regulation of photosynthesis, osmotic adjustment and synthesis of antioxidants. Casadesus et al. [97] obtained promising results in tomato plants under temperature stress, using a biostimulant based on hydrolysed animal protein, which led to increasing salicylic acid contents and favoured root growth of the treated plants. Furthermore, Liatile et al. [76] found that applying fish protein hydrolysates to *Spinacia oleracea* plants improved growth and increased chlorophyll and carotenoid content under drought conditions.

Positive effects of this type of biostimulant have also been observed under moderate or low-stress conditions; for example, Jagadeesan et al. [98] used a protein hydrolysate obtained from chicken feathers waste as biostimulant and evaluated its effect on *Zea mays* plants; they obtained significant results regarding plant growth, in addition to changes in the phytochemical profile, with increased chlorophyll, proteins and carbohydrate contents. Furthermore, Kumar et al. [99] reported that a sago bagasse hydrolysate activated the expression of genes responsible for nitrogen and carbon metabolism in *Solanum lycopersicum* plants and observed an increased germination rate and accumulation of defence metabolites. The reports mentioned above and some additional examples are summarised in Table 1.

## 5. Conclusions and Future Prospects

In this review article, we have presented a general overview of the use of plant biostimulants to improve crop growth, yields, quality traits and, especially, their defence responses to environmental abiotic stress factors in the current context of climate change. We have mainly focused on a specific type of biostimulants, those based on protein hydrolysates, generally produced from agroindustrial waste and by-products, of plant or animal origin, and on their effects on enhancing the tolerance to salt stress of horticultural crops. These biostimulants’ positive effects are currently well-established, with support from a large number of published examples, some of them mentioned in this review. However, although considerable efforts have been made to understand biostimulants’ mechanisms of action at the molecular level, they are still difficult to define specifically according to the type, origin or category of the biostimulant used. This is, therefore, a very active and interesting field of basic and applied research, and it is to be expected that many of these mechanisms will be elucidated in the near future, with the implementation of physiological, biochemical and particularly, “omics” approaches.

This review also highlights how biostimulants based on protein hydrolysates of agroindustrial waste and by-products represent a paradigmatic example of a “circular economy” and may also contribute to more sustainable agriculture by reducing the use of chemical fertilisers, in line with the EU “Green Deal” and general policies. Therefore, in addition to their proven effects as promoters of crop abiotic stress tolerance, they are also relevant from environmental, economic and social points of view, contributing to solving the problem of waste disposal or management.

## Figures and Tables

**Table 1:** Some examples of biostimulants based on protein hydrolysates of agroindustrial by-products and their most relevant effects on crops subjected to different abiotic stress treatments.

Source Material	Type of Stress	Crop	Effect on the Plants	Reference
***Agroindustrial by-products of plant origin***				
Rice and oat husks	Salt stress	Tomato (*Solanum lycopersicum* L.)	Promotes plant hydration and improves photosynthesis and primary metabolism.	[45,57,82]
		Lettuce (*Lactuca sativa* L.)	Promotes growth and protects and stimulates the absorption zone of the roots.	[89]
Graminaceae	Salt stress	Lettuce (*Lactuca sativa* L.)	Promotes growth, increasing the fresh weight of shoots.	[78]
Pumpkin seeds	Salt stress	Common bean (*Phaseolus vulgaris* L.)	Enhances the nutritional status.	[73]
Fluid agri-food vinaigrette of fruits	Salt stress	Tomato (*Solanum lycopersicum* L.)	Increased photosynthesis activity and recovery of membrane integrity.	[83]
Fabaceae or Leguminosae	Salt stress	Hemp (*Cannabis sativa* L.)	Protection of the photosynthetic system, increase in seed yield and residual biomass.	[34]
		Tomato (*Solanum lycopersicum* L.)	Improved yield and quality of fruits.	[84]
Legume seeds	Salt stress	Lettuce (*Lactuca sativa* L.)	Favours an increase in fresh yield, dry biomass and root dry weight.	[51]
	Water stress	Tomato (*Solanum lycopersicum* L.)	Favours an increase in biomass.	[53]
Legume biomass	Water stress	Grapevine (*Vitis vinifera* L.)	Promotes photosynthesis, vegetative growth and nutrient absorption.	[100]
Soybean and lupine	Water stress	Grapevine (*Vitis vinifera* L.)	Increase in soluble solids.	[86]
Sugar cane molasses with yeast extract	Drought stress	Tomato (*Solanum lycopersicum* L.)	Accumulation of antioxidant compounds, favours growth and productivity.	[101]
	Heat stress	Tomato (*Solanum lycopersicum* L.)	Favours the net photosynthetic rate and growth in plants.	[90]
Other by-products of plant origin	Chilling stress	Cucumber (*Cucumis sativus* L.)	Activation of the antioxidant enzyme system.	[102]
	Heat stress	Maize (*Zea mays* L.)	Promotes growth and stabilisation of photosynthesis.	[103]
	Metal stress	Mehirugi (*Kandelia obovate*)	Promotes the elimination of free radicals.	[104]
***Agroindustrial by-products of animal origin***				
Pig blood	Salt stress	Tomato (*Solanum lycopersicum* L.)	Promotes plant growth, chlorophyll levels and photosynthetic efficiency.	[92]
	Drought stress	Tomato (*Solanum lycopersicum* L.)	Regulates stomatal opening and promotes photosynthetic capacity.	[35]
Casein	Water stress	Grapevine (*Vitis vinifera* L.)	Increases the total anthocyanin content of the berries.	[86]
Trimmings and shavings of bovine hides	Hypoxic stress	Maize (*Zea mays* L.)	Influences the growth of roots and shoots.	[91]
Chicken feathers	Metal stress	Spinach (*Spinacia oleracea* L.)	Favours plant growth-promoting activity.	[94]
	-	Maize (*Zea mays* L.)	Promotes nutrition, yield and grain quality.	[81]
	-	Maize (*Zea mays* L.)	Promotes seed germination and plant growth.	[98]
Fish protein	Drought stress	Spinach (*Spinacia oleracea* L.)	Favours photosynthetic efficiency and growth parameters.	[76]
Fish waste (skin and scales)	-	Chilli (*Capsicum annum*)	Increases the length of shoots and roots and the number of leaves and pods.	[93]
	-	Cowpea (*Vigna unguiculata*)	Favours the formation of root nodules and fruits and increases fresh and dry weight.	[93]
Other by-products of animal origin	Salt stress	Kaki persimmon (*Diospyros kaki* L.)	Reduces chloride absorption and leaf necrosis.	[105]
	Chilling stress	Maize (*Zea mays* L.)	Increases photosynthetic activity.	[85]
	Transplant stress	Sweet pepper (*Capsicum annuum* L.)	Favours transplantation and increased yield.	[79]
	Low-temperature stress	Tomato (*Solanum lycopersicum* L.)	Promotes the growth of primary and lateral roots of plants.	[97]

## Data Availability

Not applicable.
